# Innate Immunity Evasion Strategies of Highly Pathogenic Coronaviruses: SARS-CoV, MERS-CoV, and SARS-CoV-2

**DOI:** 10.3389/fmicb.2021.770656

**Published:** 2021-10-29

**Authors:** Jin-Yan Li, Zhi-Jian Zhou, Qiong Wang, Qing-Nan He, Ming-Yi Zhao, Ye Qiu, Xing-Yi Ge

**Affiliations:** ^1^Hunan Provincial Key Laboratory of Medical Virology, Institute of Pathogen Biology and Immunology, College of Biology, Hunan University, Changsha, China; ^2^Department of Pediatrics, The Third Xiangya Hospital, Central South University, Changsha, China

**Keywords:** SARS-CoV-2, highly pathogenic coronaviruses, IFN signaling pathway, host–virus interaction, innate immunity

## Abstract

In the past two decades, coronavirus (CoV) has emerged frequently in the population. Three CoVs (SARS-CoV, MERS-CoV, SARS-CoV-2) have been identified as highly pathogenic human coronaviruses (HP-hCoVs). Particularly, the ongoing COVID-19 pandemic caused by SARS-CoV-2 warns that HP-hCoVs present a high risk to human health. Like other viruses, HP-hCoVs interact with their host cells in sophisticated manners for infection and pathogenesis. Here, we reviewed the current knowledge about the interference of HP-hCoVs in multiple cellular processes and their impacts on viral infection. HP-hCoVs employed various strategies to suppress and evade from immune response, including shielding viral RNA from recognition by pattern recognition receptors (PRRs), impairing IFN-I production, blocking the downstream pathways of IFN-I, and other evasion strategies. This summary provides a comprehensive view of the interplay between HP-hCoVs and the host cells, which is helpful to understand the mechanism of viral pathogenesis and develop antiviral therapies.

## Introduction

Coronaviruses (CoVs) are enveloped positive-sense single-stranded RNA viruses infecting various mammals and birds, including humans (Zhou et al., 2021). In the last century, human coronaviruses (hCoVs) were recognized as mild respiratory pathogens, which were barely studied regarding their low pathogenicity (Zhou et al., 2021). However, highly pathogenic hCoVs (HP-hCoVs) have emerged since the year 2003 and have caused three worldwide epidemics, including severe acute respiratory syndrome (SARS) epidemic caused by SARS-CoV in 2003, Middle East Respiratory Syndrome (MERS) epidemic caused by MERS-CoV in 2012, and the recent coronavirus disease 2019 (COVID-19) pandemic caused by SARS-CoV-2. Infections of these HP-hCoVs mainly cause acute respiratory distress syndrome (ARDS), with fatality rates of 9.5, 34.4, and 2.3% for SARS-CoV, MERS-CoV, and SARS-CoV-2, respectively (Petrosillo et al., 2020). Especially, SARS-CoV-2 shows the highest transmissibility among the three HP-hCoVs, which has led to one of the most severe global pandemics with 213 million infected cases and over 4.4 million deaths as of August 23, 2021 (WHO, n.d.). An overwhelming preponderance of cases and deaths is reported in the elderly, especially with underlying diabetes, cardiovascular, and hypertension comorbidities. By contrast, few severe cases are found in young children, whose innate immunity response is highly effective (Bajaj et al., 2020). Innate immunity is a determinant factor for disease outcome. Although 4,680 million vaccine doses have been administered, confirmed cases of COVID-19 are increasing sharply, drawing more and more attention of researchers around the world on the pathogenesis of HP-hCoV infection (WHO, n.d.). Pathogenesis is determined by the interplay between HP-hCoV and host antiviral defense. This review summarized the innate immunity evasion tactics employed by HP-hCoVs, focusing on the interactions of various viral proteins and host signaling pathways.

## Highly Pathogenic Human Coronaviruses and the IFN System

**FIGURE 1:**
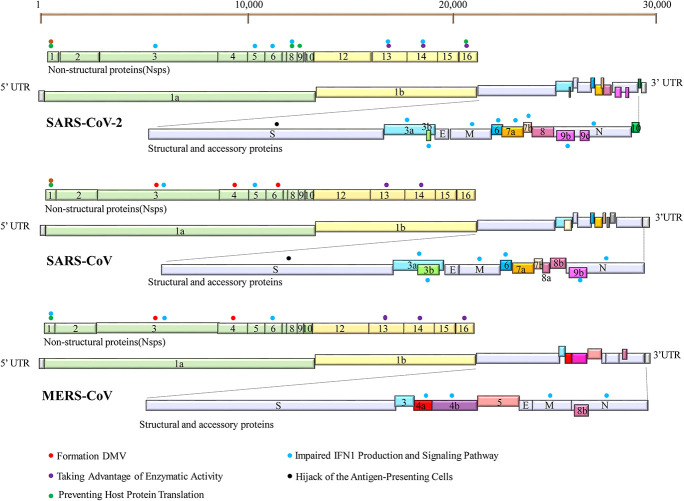
Genome organization of SARS-CoV-2, SARS-CoV, and MERS-CoV. The common trait is that their genomes encode two replicase polypeptides pp1a and pp1b translated from ORF1a and ORF1b. The polypeptides undergo a series of proteolytic cleavages to form 16 non-structural proteins encoded by the first two-thirds of the genome. The remaining one-third 3′ region of the viral genome encodes viral structural proteins [spike (S), membrane (M), envelope (E), and nucleocapsid (N) proteins] and virus-specific accessory proteins. Accessory proteins are interspersed within these structural proteins. Some proteins can inhibit innate immune responses by employing a variety of tactics. Their locations are indicated with specific-colored spheres. The different colored spheres represent the different immune evasive strategies. Each virus-encoded multifunctional protein, employing multiple different activities to suppress innate immunity responses.

All the three HP-hCoVs are single-stranded RNA (ssRNA) viruses classified into *Betacoronaviruses* genus of *Orthocoronavirinae* subfamily in *Coronaviridae* family (Group, 2020; Zhou et al., 2021). Specifically, SARS-CoV and SARS-CoV-2 belong to the subgenus of *Sarbecovirus*, while MERS-CoV is classified into the subgenus of *Merbecovirus* (Zhou et al., 2021). The SARS-CoV-2 genome shares around 79% identity with SARS-CoV and 30% with MERS-CoV, respectively (Zhou et al., 2020). Their typical genomic organization contains non-structural, structural, and accessory proteins flanked by a 5′-cap structure and a 3′-poly (A) tail (Yang and Leibowitz, 2015). The open reading frame (ORF) 1a and ORF1b occupy the two-third 5′ region of the viral genome and can be directly translated into two large polyproteins, pp1a and pp1b, which are further hydrolyzed into 16 non-structural proteins (NSP1 ∼ NSP16) by two viral proteases, NSP3 (papain-like protease, PL-pro) and NSP5 (3C-like protease, 3CL-pro) (Zhou et al., 2021). These NSPs take shape the replication-transcription complex (RTC), which is necessary for viral RNA transcription and replication (Li et al., 2020b). NSPs of different CoVs are evolutionarily conservative except for NSP1 and NSP2, usually virulent factors. The remaining one-third 3′ region of the viral genome encodes viral structural proteins (S, E, M, and N) and virus-specific accessory proteins, which are translated from the subgenomic RNAs (sgRNAs) synthesized in the discontinuous viral transcription process (Zhang Z. et al., 2021). Some of these ORFs are overlapping or found within a larger ORF (Zhou et al., 2021). Accessory proteins are distinct for different CoVs in their numbers, sequences, genomic locations, and functions. For instance, nine accessory proteins (ORF3a, ORF3b, ORF6, ORF7a, ORF7b, ORF8, ORF9b, ORF9c, and ORF10) of SARS-CoV-2, eight accessory proteins (ORF3a, ORF3b, ORF6, ORF7a, ORF7b, ORF8a, ORF8b, and ORF9b) of SARS-CoV, and five accessory proteins (ORF3a, ORF4a, ORF4b, ORF5, and ORF8b) of MERS-CoV have been identified (Figure 1). Among these proteins, many have been reported to suppress the innate immunity against HP-hCoV infection, which is considered critical for the pathogenesis of HP-hCoVs.

**FIGURE 2:**
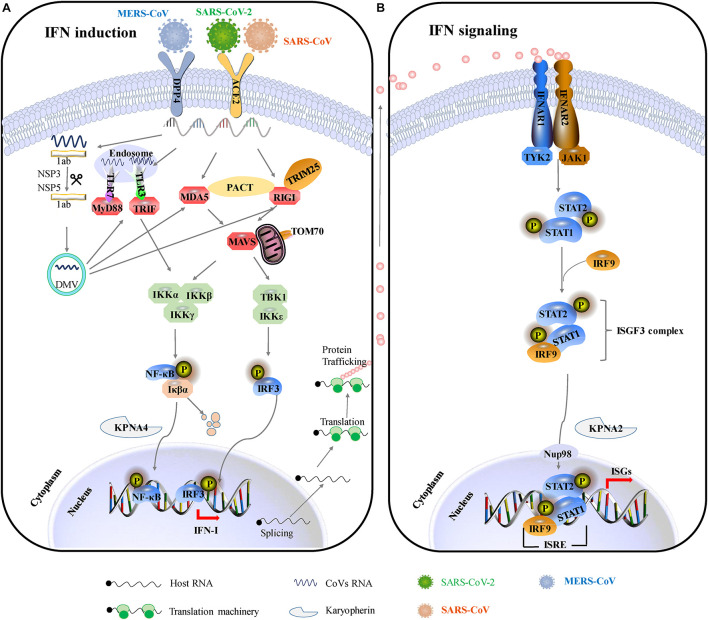
IFN-mediated antiviral responses. **(A)** SARS-CoV and SARS-CoV-2 use the same receptor ACE2 for host cellular entry, while MERS-CoV utilizes DPP4 for host cellular entry. They are most likely recognized by PRRs, including cytoplasmic RIG-I and MDA5 and by endosomal TLR3 and TLR7. RIG-I/MDA5 conveys signal through a mitochondrial adaptor MAVS, while TLR signals through TRIF/MyD88. Subsequently, activating the shared downstream kinases and transcription factors. Both pathways can activate the key transcription factors (IRF3 and NF-κB) phosphorylation and subsequent dimerization, which are translocated into the nucleus to promote IFN-I expression. **(B)** IFNs bind to the heterodimeric receptor complexes IFNAR1/IFNAR2, initiating JAK/STAT signaling. Receptor-associated tyrosine kinases Jak1 and Tyk2 are triggered for self-phosphorylation and activation, leading to the phosphorylation of STATs. Phosphorylated STAT1/2 with IRF9 forms a complex ternary ISGF3 (STAT1/STAT2/IRF9), which translocates into the nucleus and binds to IFN-stimulated response elements (ISREs), promoting the transcription of hundreds of IFN-stimulated genes (ISGs). ACE2, angiotensin-converting enzyme 2; DPP4, dipeptidyl peptidase 4; DMVs, double-membraned vesicles; RIG-1, retinoic acid-inducible gene I; MDA5, melanoma differentiation-associated protein 5; MyD88, myeloid differentiation primary response 88; TRIF, TIR-domain-containing adapter-inducing IFN-β; MAVS, mitochondria antiviral signaling protein; PACT, protein activator of protein kinase R; Iκβα, inhibitor of NF-κB; IRF3, interferon regulatory factor 3; TBK1, TANK-binding kinase 1; IKKε, I-kappa-B kinase ε; Nup93, nuclear pore complex protein 93; KPNA4, karyopherin-α4; KPNA2, karyopherin-α2; ISGF3, IFN-stimulated gene factor 3; IFNAR1, interferon-alpha/beta receptor alpha chain KPNA2, karyopherin-α2; ISG, interferon gene expression; JAK/STAT, Janus kinases/signal transducer and activator transcription proteins.

The innate immunity acts as a frontline of defense characterized mainly by type I interferon (IFN-I) response, including recognizing pathogen-associated molecular patterns (PAMPs), IFN induction, and IFN signal transduction (Figure 2). Upon CoV infection, viral RNA could be recognized by the RIG-I-like receptors (RLRs), including retinoic acid-inducible gene I (RIG-I), melanoma differentiation gene 5 (MDA5), and toll-like receptors (TLRs) (Ivashkiv and Donlin, 2014). RIG-I and MDA5 trigger the downstream adaptor mitochondrial antiviral signaling protein (MAVS) on mitochondria. MAVS subsequently recruits the two IKKε and TANK-binding kinase 1 (TBK1), leading to phosphorylation and nuclear translocation of IFN-regulatory factor 3 (IRF3), which induces the expression and secretion of IFNs (Kawai et al., 2005). In addition, MAVS recruits IKK-related kinases (IKKα, IKKβ, and IKKγ) and activates the NF-κB pathway by promoting phosphorylation and nuclear translocation of p65, leading to cytokine production (Kawai et al., 2005). The secreted IFN-I can turn on the antiviral status in infected or neighboring cells through autocrine or paracrine. In brief, once the secreted IFN-I bind to their receptors, IFNAR (interferon alpha/beta receptor), on the cell surface, the downstream Janus kinase (Jak)/signal transducer and activator of transcription (STAT) signal pathway will be initiated by the activation of receptor-associated Jak1/TYK2 (tyrosine kinase 2). Then, phosphorylated STAT1 and STAT2 form heterodimers, interacting with IFN regulatory factor 9 (IRF9) to form an IFN-stimulated gene factor 3 (ISGF3) transcription complex, which translocates to the nucleus and binds to IFN-stimulated response elements (ISREs) in gene promoters, thereby activating the expression of interferon-stimulated genes (ISGs) to establish the host antiviral status (Ivashkiv and Donlin, 2014). Therefore, IFN-I is a strong immune modulator. It has a wide range of antiviral functions: (1) it can induce the expression of various antiviral proteins and restrict the synthesis of viral proteins, thus impairing virus replication; (2) it can induce apoptosis of infected cells, eliminating the “virus production factory”; (3) it can promote the maturation and activation of dendritic cells, promoting the activation of the adaptive immune response.

**FIGURE 3:**
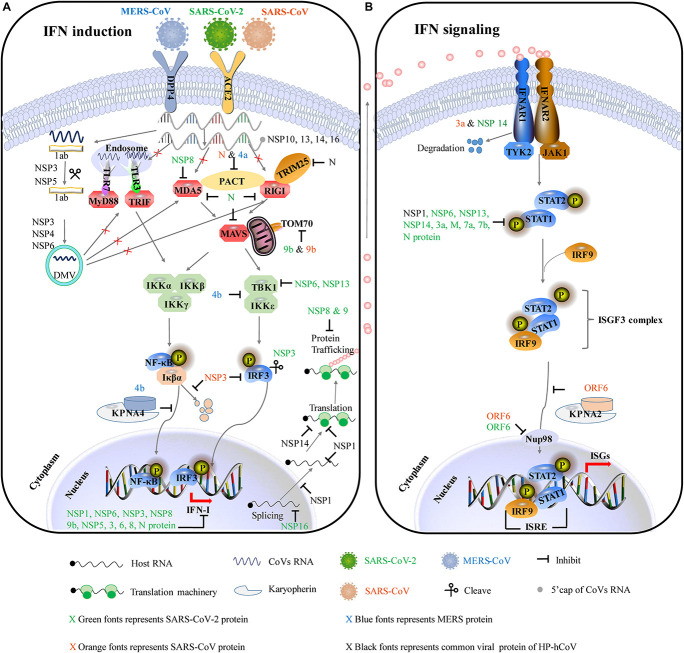
The major immune evasive strategies by HP-hCoVs as discussed in this review. The CoV-encoded proteins inhibit multiple aspects of the host innate immune signaling from sustaining viral replication and propagation. Different colors represent the proteins encoded by different viruses. Green represents SARS-CoV-2 encoded proteins, orange represents SARS-CoV encoded proteins, blue represents MERS-CoV encoded proteins, and black represents their common proteins. Virus antagonistic tactics are shown with black lines and arrows. **(A)** HP-hCoVs uses multiple gene products to impair IFN induction. To shield viral RNA (ssRNA and dsRNA) from recognition by PRRs, CoV replication takes place and DMVs are formed by NSP3, NSP4, and NSP6. In addition, NSP10, NSP13, NSP14, and NSP16 can modify the 5′-cap structure of viral RNA to mask viral PAMPs. HP-hCoVs interfere with the transmission of signals at almost every step. HP-hCoV N proteins interact with TRIM25, interfering with RIG-I signaling. MERS-CoV 4a and SARS-CoV N associate with PACT, sequestering the association of PACT and RIG-I/MDA5. SARS-CoV-2 NSP8 interacts with MDA5 to interfere with IFN induction. SARS-CoV-2 N is reported to associate with RIG-I, MDA5, and MAVS. SARS-CoV-2 and SARS-CoV M proteins are reported to associate with multiple adapters (not shown). SARS-CoV-2 and SARS-CoV 9b proteins interact with human TOM70 to block signaling downstream of MAVS. MERS-CoV ORF4b could specifically interact with TBK1 and IKKε, thereby blocking IRF3 phosphorylation. In addition, MERS-CoV ORF4b can associate karyopherin-α4 (KPNA4), out-competing NF-κB for KPNA4 binding and suppressing NF-κB nuclear transport. SARS-CoV NSP3 binds to IRF3 and inhibits the degradation of IκBα. SARS-CoV-2 NSP3 can cleave IRF3 directly. HP-hCoV NSP1 efficiently interferes with the cellular translation machinery. SARS-CoV-2 NSP16 disrupts mRNA splicing. SARS-CoV-2 NSP8 and NSP9 interfere with host protein trafficking. **(B)** HP-hCoVs use multiple gene products to impair IFN signaling. SARS-CoV 3a and SARS-CoV-2 NSP14 can degrade IFNAR1. SARS-CoV and SARS-CoV-2 ORF6 bind directly to Nup98 and Rae1 to prevent bidirectional nucleocytoplasmic transport. SARS-CoV ORF6 associates karyopherin-α2 (KPNA2), retaining KPNA2 in the cytoplasm and suppressing STAT1 nuclear import. SARS-CoV-2 NSP6, NSP13, NSP14, 3a, M, 7a, 7b, and N protein inhibit the phosphorylation of STAT1. HP-hCoV NSP1 inhibits the phosphorylation of STAT.

Based on previous therapeutic interventions of IFNs during SARS-CoV or MERS-CoV infection, IFN-β was the most potent IFN-I subtype (Antonelli et al., 2003; Hensley et al., 2004; Omrani et al., 2014). Compared to SARS-CoV and MERS-CoV, SARS-CoV-2 is substantially more sensitive to IFN-I. IFNs do alleviate the pathogenesis of those HP-hCoV-infected patients. Two therapeutic regimens of IFN-α + lopinavir/ritonavir and IFN-α + lopinavir/ritonavir + ribavirin are beneficial for COVID-19 patients (Yuan et al., 2020). Inhaled nebulized interferon beta-1a (SNG001) has greater odds of improvement, and treated patients recovered more rapidly from SARS-CoV-2 infection (Monk et al., 2021). Numerous independent clinical trials confirm that IFN therapy could attenuate the clinical consequences of COVID-19 in the early stages of infection (Sallard et al., 2020). These facts strongly indicate that impaired IFN-I expression may at least partially contribute to the severity of the disease. Indeed, CoVs do counter the IFN-I response by employing multipronged strategies to survive in the host. Given the importance of IFN-I responses in the pathogenesis of HP-hCoVs, the mechanisms of these viruses to antagonize IFN-I responses are discussed in more detail below and graphical summary is shown in Figure 3.

## Shielding Viral RNA From Recognition by Pattern Recognition Receptors

Protecting the ‘self’ from the ‘non-self’ is an important part of innate immunity. Upon CoV infection, distinguishing cellular RNA from external RNA is essential for living organisms to maintain life. Although organisms employ a well-developed mechanism for recognizing harmful external factors, HP-hCoVs have evolved three means to evade detection by the innate immunity RNA sensors: (1) double-membrane vesicles (DMV) hiding nascent viral RNAs from PRRs; (2) modification of the 5′-cap structure to mask viral PAMPs; (3) manipulation of stress granule formation. HP-hCoVs utilize the above diverse strategies to avoid their RNA recognition by PRRs, concomitantly guaranteeing their mRNAs to be recognized felicitously by the translation machinery of the host (Table 1).

**TABLE 1:** Strategies of highly pathogenic human coronaviruses (HP-hCoVs) to shield viral RNA from recognition by pattern recognition receptors (PRRs).

**Protein**	**Virus**	**Mechanism**	**References**
**DMV hiding nascent viral RNAs from PRRs**			
NSP3, NSP4, and NSP6	SARS-CoV, MERS-CoV	Formation DMV to hide nascent viral RNAs from PRRs	Angelini et al., 2013; Oudshoorn et al., 2017; Klein et al., 2020
**Modification of the 5′-Cap structure to mask viral PAMPs**			
NSP10, NSP13, NSP14, and NSP16	HP-hCoVs	Mediating mRNA capping	von Grotthuss et al., 2003; Chen et al., 2009; Chang and Chen, 2021; Gorkhali et al., 2021; Ramasamy and Subbian, 2021
**Manipulation of stress granule formation (SGs)**			
4a	MERS-CoV	Preventing SG formation by binding to viral dsRNA and impairing dsRNA-mediated PKR activation	Nakagawa et al., 2018
NSP15	SARS-CoV-2, SARS-CoV	Antagonizing the formation of chemical-induced SGs	Gao et al., 2021
N	SARS-CoV-2, SARS-CoV	Impairing dsRNA-triggered SG formation by associating with the PKR and G3BP1	Gordon et al., 2020; Zheng et al., 2021
	MERS-CoV	Blocking SG formation by interacting with PKR but not G3BP1	Zheng et al., 2021

### Double-Membrane Vesicles Hiding Nascent Viral RNAs From Pattern Recognition Receptors

The replication of CoV RNA is processed at cytoplasmic membranous replication organelles (ROs). DMVs are a prominent type of virus-related ROs. CoV infection induces host endoplasmic reticulum (ER) alterations and rearranges, leading to the formation of DMVs, which shield the nascent viral RNAs from recognition by cytosolic PRRs such as RIG-I/MDA5 and endosomal PRRs such as TLR3/TLR7 (Taefehshokr et al., 2020). SARS-CoV-2 infection induces an intense membrane remodeling and forms the double-lipid bilayer in electron micrographs (Klein et al., 2020). Surprisingly, a triple transfection of NSP3, NSP4, and NSP6 of SARS yielded DMV, similar to those induced in SARS coronavirus infected cells (Angelini et al., 2013). Similarly, co-expression of MERS-CoV NSP3 and NSP4 either as individual proteins or as a self-cleaving NSP3-4 precursor induced the formation of DMVs, whereas MERS-CoV NSP6 did not noticeably affect DMV formation (Oudshoorn et al., 2017).

### Modification of the 5′-Cap Structure to Mask Viral Pathogen-Associated Molecular Patterns

The genomes of HP-hCoVs contain the standard eukaryotic 5′-terminal 7-methylguanosine cap structure and a 3′ poly (A) tail, mimicking cellular mRNA to shield the pathogen-associated molecular patterns (PAMP) on the viral genome from the recognition of PRRs. The 5′ capping of eukaryotic mRNA is an important strategy to distinguish cellular RNA from external RNA. Uncapped viral RNAs could be recognized by a series of PRRs, initiating host immune response. To mimic eukaryotic RNA structures and evade the recognition by PRRs, CoVs process post-translational capping of viral mRNA catalyzed by the capping enzymes in their polymerase complexes, including NSP13 functions as RNA helicase and 5′-triphosphatase, NSP14 as an exoribonuclease and N7-MTase, and NSP16 as a 2′-*O* methyltransferase (2′-*O*-MTase) (Chang and Chen, 2021). The 2′-*O*-methyltransferase function is associated with K-D-K-E (lysine-aspartate-lysine-glutamate) motif in NSP13 conserved among all HP-hCoVs (von Grotthuss et al., 2003). In addition, NSP14 of HP-hCoVs possesses guanine N7-MTase activity coupled with exonuclease activity, involving RNA cap formation and cleaving RNA-PAMPs, also contributing to immune evasion (Chen et al., 2009; Ramasamy and Subbian, 2021). NSP16 of all HP-hCoVs, with its activating cofactor nsp10, can form a 2′-*O*-methylated cap for immune evasion in which the conserved D130 is critical (Gorkhali et al., 2021). Taken together, the 5′ cap modification of viral RNA impairs the recognition by cytosolic PRRs, thereby resisting the IFN-mediated antiviral response. The functions of polymerase complexes are common to CoVs.

### Manipulation of Stress Granule Formation

Stress granules (SGs) are membraneless cytoplasmic RNA granules responding to various stresses, including virus infection. Upon virus infection, the host can form SGs by wrapping viral RNA, transcription and translation-related proteins. The accumulation of viral RNA provides a pool of substrates for PRRs such as RIG-I and MDA5, triggering RIG-I like receptor signaling pathway (Onomoto et al., 2012; Kim et al., 2019; Kikkert, 2020). Indeed, SGs form a platform for innate immunity and play a significant role in antiviral response. However, HP-hCoVs can inhibit the formation of SGs to antagonize innate immunity responses for benefiting virus replication. MERS-CoV encoded 4a protein could prevent stress granule formation by binding to viral dsRNA and impairing dsRNA-mediated PKR (protein kinase R) activation, promoting viral translation and virus replication (Nakagawa et al., 2018). In addition, NSP15 from SARS-CoV and SARS-CoV-2, harboring the conserved function, can antagonize the formation of chemical-induced SGs (Gao et al., 2021). SARS-CoV-2 and SARS-CoV N proteins could associate with the protein kinase PKR and stress granule protein G3BP1 (the Ras-GTPase-activating protein SH3 domain-binding protein 1), impairing dsRNA-triggered SG formation (Gordon et al., 2020; Zheng et al., 2021). In contrast, MERS-CoV N can associate with PKR but not G3BP1 to block SG formation (Zheng et al., 2021).

## Impaired IFN-I Production and Signaling Pathway

Dysregulated IFN-I responses contribute to the robust early HP-hCoV replication and trigger a cytokine storm. The HP-hCoVs employ extensive measures to interfere with the key host signaling factors to counteract the IFN system by fully using certain proteins, including accessory proteins, NSPs, and structural proteins. Recent studies report that SARS-CoV-2 NSP1, NSP3, NSP6, NSP12, NSP13, NSP14, NSP15, M, ORF3a, ORF3b, ORF6, ORF7a, ORF7b, ORF9b, and N can interfere with the key host signaling factors of IFN signaling pathway. It has been documented that more than half of SARS-CoV-2 proteins have antagonistic effects on IFN responses by either targeting viral sensors or blocking downstream antiviral signaling molecules. The detailed antagonistic effects on IFN responses by NSPs, accessory proteins, and structural proteins are summarized one by one below (Table 2).

**TABLE 2:** Evasion IFN-I system by HP-hCoV-encoded proteins.

**Protein**	**Virus**	**Mechanism**	**References**
**Non-structural proteins**			
NSP1	HP-hCoVs	Suppressing phosphorylation of STAT1 and STAT2	Xia et al., 2020; Kumar et al., 2021
NSP3	SARS-CoV-2	Cleaving IRF3 directly	Moustaqil et al., 2021
	SARS-CoV-2, SARS-CoV,	Cleaving ubiquitin-like protein ISG15	Shin et al., 2020
	HP-hCoVs	Exhibiting DUB and deISGylating activities	Ratia et al., 2014; Yang et al., 2014; Shin et al., 2020
	SARS-CoV	Binding to IRF3 and inhibiting the degradation of IκBα	Devaraj et al., 2007; Frieman et al., 2009
	MERS-CoV	Blocking IRF3 phosphorylation and nuclear transport	Yang et al., 2014
NSP5	SARS-CoV-2, SARS-CoV	Preventing nuclear translocation of phosphorylated IRF3	Fung et al., 2021
NSP6	SARS-CoV-2, MERS-CoV	Binding to TBK1 and suppressing IRF3 phosphorylation	Xia et al., 2020
NSP8	SARS-CoV-2	Binding to MDA5 and impairing its K63-linked polyubiquitination	Yang et al., 2020
NSP13	SARS-CoV-2	Binding and blocking TBK1 phosphorylation	Yuen et al., 2020
NSP14	SARS-CoV-2	Inducing lysosomal degradation of the IFNAR1 (IFN-I receptor) and inhibiting STAT activation	Hayn et al., 2021
**Accessory proteins**			
ORF3a	SARS-CoV-2	Impeding the phosphorylation of STAT1	Lei et al., 2020; Xia et al., 2020
	SARS-CoV	Degradation of IFNAR1	Minakshi et al., 2009
ORF3b	SARS-CoV-2, SARS-CoV	Hampering the nuclear translocation of IRF3	Konno et al., 2020
ORF6	SARS-CoV-2, SARS-CoV	Binding directly to the Nup98 and Rae1 to Prevent bidirectional nucleocytoplasmic transport	Kopecky-Bromberg et al., 2007; Addetia et al., 2021
	SARS-CoV	Tethering KPNA2 and suppressing nuclear translocation of STAT1	Frieman et al., 2007
ORF7a	SARS-CoV-2	Impeding phosphorylation of STAT1 but STAT2	Xia et al., 2020; Suryawanshi et al., 2021
ORF7b	SARS-CoV-2	Suppressing phosphorylation of STAT1 and STAT2	Xia et al., 2020; Suryawanshi et al., 2021
ORF8	SARS-CoV-2	Attenuating SeV induced IFN-β promoter activation and IFN-β mRNA level	Li et al., 2020a
ORF9b	SARS-CoV-2	Interrupting K63-linked ubiquitination of NEMO	Wu J. et al., 2021
	SARS-CoV-2, SARS-CoV	Interacting with human TOM70	Jiang et al., 2020; Thorne et al., 2021
	SARS-CoV	Targeting MAVS by usurping poly(C)-binding protein 2 (PCBP2) and the HECT domain E3 ligase AIP4	Shi et al., 2014
ORF4a	MERS-CoV	Binding to PACT	Siu et al., 2014
ORF4b	MERS-CoV	Interacting with TBK1 and IKKε,	Yang et al., 2015
		Associating with KPNA4 and suppressing nuclear translocation of NF-κB	Canton et al., 2018
**Structural proteins**			
M	SARS-CoV-2	Interacting with MAVS	Fu et al., 2021
		Associating with MDA5, TRAF3, IKK**ℰ**, and TBK1 and degrading TBK1 *via* the ubiquitin pathway	Sui et al., 2021
	SARS-CoV	Associating with RIG-I, TBK1, IKKε, and TRAF3 and impedes the formation of TRAF3/TANK/TBK1 complex	Siu et al., 2009
	MERS-CoV	Interacting with TRAF3	Lui et al., 2016
N	SARS-CoV-2	Interacting with MDA5 and RIG-I and blocking the IRF3 phosphorylation and nuclear translocation	Chen et al., 2020
	SARS-CoV-2	Blocking the STAT1 and STAT2 phosphorylation and nuclear translocation	Mu et al., 2020
	SARS-CoV-2	Inhibiting Lys63-linked poly-ubiquitination and aggregation of MAVS	Wang S. et al., 2021
	HP-hCoVs	Interfering with TRIM25-mediated RIG-I ubiquitination	Hu et al., 2017; Chang et al., 2020; Gori Savellini et al., 2021
	SARS-CoV	Binding with PACT	Ding et al., 2017

### Non-structural Proteins

The CoVs NSPs have multiple functions, including viral transcription and replication and antagonizing IFN responses. SARS-CoV-2 NSP1 has been confirmed to block IRF3 phosphorylation and nuclear transport. Moreover, SARS-CoV-2 NSP1 causes the depletion of antiviral factors Tyk2 and STAT2, showing a decrease in STAT1 and STAT2 phosphorylation (Kumar et al., 2021). SARS-CoV-2 NSP1 owns 84% amino acid sequence identity with SARS-CoV NSP1, showing a similar mechanism to antagonize IFN-I response. Furthermore, comparing with SARS-CoV and MERS-CoV NSP1, SARS-CoV-2 NSP1 suppresses phosphorylation of STAT1 and STAT2 more efficiently (Xia et al., 2020). The CoV proteases NSP3 and NSP5 are multifunctional proteins, playing an essential role in the proteolytic processing of the viral polyproteins, maturation, and assembly of the RTC and cleaving proteinaceous post-translational modifications on host proteins. The cleavage of innate immunity factors, antagonizing IFN and its downstream JAK-STAT signal transduction, is a particularly effective strategy to evade the host innate immunity.

SARS-CoV-2 NSP3 directly cleaves IRF3, resulting in reduced IFN production. SARS-CoV and SARS-CoV-2 NSP3 cleave ubiquitin-like protein ISG15, a typical regulator of host innate immune signaling pathways, decreasing IFN production and enhancing virus replication and spread (Moustaqil et al., 2021). Compared with SARS-CoV NSP3, SARS-CoV-2 NSP3 preferentially reduces ISG15-conjugated (ISGylated) protein substrates (Shin et al., 2020). Besides proteolytic activity, CoVs NSP3 acts as a deubiquitinating enzyme (DUB) processing deISGylating activity, efficiently degrading mono-polyubiquitin, di-polyubiquitin, and branched-polyubiquitin chains. The deubiquitinating modifications from host cell proteins disturb the host innate immune responses to viral infection (Lindner et al., 2007; Ratia et al., 2014; Baez-Santos et al., 2015; Clasman et al., 2020). SARS-CoV-2 NSP3 has 83% sequence identity with SARS-CoV NSP3 but exhibits different host substrate preferences. SARS-CoV-2 NSP3 preferentially cleaves the ubiquitin-like interferon-stimulated 15 protein (ISG15), whereas SARS-CoV NSP3 predominantly targets ubiquitin chains (Shin et al., 2020). SARS NSP3 removes Lys63-linked ubiquitin chains of TRAF3 and TRAF6, but not Lys48-linked ubiquitin chains, suppressing the Toll-like receptor 7 (TLR7) signaling pathway (Li et al., 2016). Similarly, MERS-CoV NSP3 exhibits DUB activity and acts on both K48- and K63-linked ubiquitination and ISG15-linked ISGylation, and the catalytic sites C1592 and H1759 are required for deISGylation (Yang et al., 2014). Expect for cleavage and deISGylating activities, HP-hCoV NSP3 could interfere with the signaling factors of the interferon signaling pathway. SARS-CoV NSP3 interacts with STING (stimulator of interferon genes, also known as MITA/ERIS/MYPS), inhibiting the phosphorylation and dimerization of IRF3 (Sun et al., 2012; Chen et al., 2014; Matthews et al., 2014).

In addition, SARS-CoV NSP3 can associate with IRF3 and block the phosphorylation and nuclear translocation of IRF3 (Devaraj et al., 2007). Moreover, SARS-CoV Nsp3 can inhibit the degradation of IκBα, an inhibitor of NF-κB, suppressing the NF-κB signaling pathway (Frieman et al., 2009). MERS-CoV NSP3 acts as an IFN antagonist by blocking the phosphorylation and nuclear translocation of IFN regulatory factor 3 (IRF3) (Yang et al., 2014). Confirmed by the above, CoV NSP3 is a multifunctional protein, so is CoVs NSP5. NSP5 proteins of SARS-CoV and SARS-CoV-2, sharing 96% amino acid sequence identity, suppress IFN-I production by perturbing nuclear translocation of phosphorylated IRF3 without affecting the phosphorylation of IRF3 (Fung et al., 2021).

SARS-CoV-2 NSP6 associates with TANK binding kinase 1 (TBK1) without affecting TBK1 phosphorylation, and the NSP6/TBK1 interaction suppresses IRF3 phosphorylation (Xia et al., 2020). SARS-CoV-2 NSP6 inhibits IFN-I production more efficiently than MERS-CoV NSP6. However, SARS-CoV NSP6 does not inhibit the IFN-I response (Xia et al., 2020). SARS-CoV-2 NSP8 inhibits the expression of IFN-I, IFN-stimulated genes, and proinflammatory cytokines by associating with MDA5 and impairing its K63-linked polyubiquitination (Yang et al., 2020). SARS-CoV-2 NSP12, the viral RNA-dependent RNA polymerase (RdRp), attenuates SeV or Poly(I:C) induced IFN-β promoter activation by suppressing the nuclear translocation of IRF3 but does not impair IRF3 phosphorylation (Wang W. et al., 2021). Paradoxically, another article reported that SARS-CoV-2 NSP12 protein is not an IFN-β antagonist, owing to NSP12 inhibits neither IFN-β production nor downstream IFN-β signaling pathway (Li et al., 2021). SARS-CoV-2 NSP13 binds and blocks TBK1 phosphorylation, decreasing IRF3 activation (Yuen et al., 2020). Moreover, SARS-CoV-2 NSP13 can suppress IFN-I signaling by inhibiting STAT1 and STAT2 activation, leading to the retention of STAT1 in the cytoplasm and compromising stimulation of the ISRE promoter (Lei et al., 2020). SARS-CoV-2 NSP14 induces lysosomal degradation of the IFNAR1, thereby inhibiting STAT activation (Hayn et al., 2021).

### Accessory Proteins

Accessory proteins of CoVs are not required for replication, but they play critical roles during infection and pathogenesis, owing to antagonizing the host response. Different studies have reported that SARS-CoV-2 ORF3a inhibits IFN signaling by impeding STAT1 phosphorylation (Lei et al., 2020; Xia et al., 2020). At the same time, SARS-CoV ORF3a induces the degradation of IFNAR1 to increase IFNAR1 ubiquitination (Minakshi et al., 2009). SARS-CoV-2 ORF3b has only 22 amino acids (69 bp, including the stop codon), which could inhibit the induction of IFN more efficiently than its SARS-CoV ortholog (153 amino acids on average), and its anti-IFN activity is increased by a naturally occurring elongation variant (Konno et al., 2020). SARS-CoV and SARS-CoV-2 ORF6 bind directly to Nup98 and Rae1 to prevent bidirectional nucleocytoplasmic transport, namely, nuclear export of host mRNA and nuclear import of various host factors, blocking IRF3 and STAT nuclear import (Kopecky-Bromberg et al., 2007; Addetia et al., 2021). Moreover, compared to SARS-CoV ORF6, SARS-CoV-2 ORF6 may more dramatically suppress protein expression through a stronger interaction with the Rae1and Nup98 (Addetia et al., 2021). Consistently, another study confirmed that SARS-CoV-2 ORF6 causes the accumulation of heterogeneous ribonucleoprotein A (hnRNPA1) in the nucleus (Kato et al., 2021).

In line with the above result, SARS-CoV-2 ORF6 interferes less efficiently with human interferon induction and interferon signaling than SARS-CoV ORF6 using reverse genetics (Schroeder et al., 2021). SARS-CoV ORF6 can associate karyopherin-α2 (KPNA2), retaining KPNA2 in the cytoplasm, and suppressing STAT1 nuclear import. Furthermore, recombinant SARS-CoV lacking ORF6 did not tether KPNA2 to the ER/Golgi membrane and allowed the import of the STAT1 complex into the nucleus (Frieman et al., 2007). SARS-CoV-2 ORF7a could impede the phosphorylation of STAT1 but STAT2, while SARS-CoV-2 ORF7b could suppress STAT1 and STAT2 phosphorylation (Xia et al., 2020; Suryawanshi et al., 2021). SARS-CoV-2 ORF8 can attenuate SeV induced IFN-β promoter activation and IFN-β mRNA level (Li et al., 2020a).

SARS-CoV-2 ORF9b has been reported to impede the host innate immune system by targeting multiple molecules. SARS-CoV-2 ORF9b interrupts K63-linked ubiquitination of NEMO upon VSV stimulation, thereby inhibiting the canonical IκB kinase alpha (IKKα)/β/γ-NF-κB signaling and subsequent IFN production (Wu J. et al., 2021). SARS-CoV-2 and SARS-CoV ORF9b block signaling downstream of MAVS by interacting with human TOM70, and that this process is regulated by phosphorylation (Jiang et al., 2020; Thorne et al., 2021). SARS-CoV ORF9b targets MAVS by usurping poly(C)-binding protein 2 (PCBP2) and the HECT domain E3 ligase AIP4, leading to the degradation of MAVS, TRAF3, and TRAF6 (Shi et al., 2014). MERS-CoV ORF4a protein binds with protein kinase R (PACT) protein activator, thereby inhibiting PACT-induced activation of RIG-I and MDA5 (Siu et al., 2014). MERS-CoV ORF4b could specifically interact with TBK1 and IKKε, thereby blocking IRF3 phosphorylation (Yang et al., 2015). In addition, MERS-CoV ORF4b can bind to karyopherin-α4 (KPNA4), out-competing NF-κB for KPNA4 binding and suppressing NF-κB nuclear transport (Canton et al., 2018).

### Structural Proteins

Coronaviruses structural proteins are mainly responsible for viral assembly, coating, entry into host cells, and packaging of the RNA genome (Endriyas Kelta et al., 2020). In addition, M and N proteins can block the IFN-I response. CoV M protein is a glycosylated structural protein with three membrane-spanning domains. M protein predominantly localizes to the Golgi complex and is necessary for the assembly of viral particles. SARS-CoV-2 M, blocking RIG-I and MAVS, but not TBK1, IKKε, and IRF3-5D triggered IFN-β promoter activation, inhibits the innate antiviral response by interacting with MAVS, and its TM1/2 domains are essential for this inhibitory function (Fu et al., 2021). Paradoxically, another study reported that SARS-CoV-2 M associates with MDA5, TRAF3, IKKℰ, and TBK1 and degrades TBK1 *via* ubiquitin pathway (Sui et al., 2021). However, the SARS-CoV M protein physically associates with RIG-I, TBK1, IKKε, and TRAF3 and impedes the formation of TRAF3/TANK/TBK1 complex (Siu et al., 2009), whereas the MERS CoV M protein interacts with TRAF3 and disrupts TRAF3-TBK1 association, leading to reduced phosphorylation of IRF3 (Lui et al., 2016).

HP-hCoVs M proteins collectively possess the common and conserved mechanism of IFN-I expression attenuation, but they target different signal adapters. SARS-CoV-2 N protein is confirmed as the interferon antagonist by different research teams, targeting multiple factors. It interacts with MDA5 and RIG-I through the DExD/H domain of RIG-I, leading to the IRF3 phosphorylation and nuclear translocation and STAT1 and STAT2 (Chen et al., 2020; Mu et al., 2020). Besides, SARS-CoV-2 N protein, undergoing liquid–liquid phase separation with RNA, inhibits Lys63-linked poly-ubiquitination and aggregation of MAVS and thereby suppresses the innate antiviral immune response (Wang S. et al., 2021). SARS-CoV-2 N protein targets the initial step and interferes with TRIM25-mediated RIG-I ubiquitination (Gori Savellini et al., 2021). Like SARS-CoV-2 N protein, SARS-CoV and MERS-CoV N proteins interfere with RIG-I signaling by interacting with TRIM25 (Hu et al., 2017; Chang et al., 2020). Another literature reported that SARS-CoV N protein could associate with protein activator of protein kinase R (PACT), activating RIG-I and MDA5. The N-PACT association sequestered the binding between PACT and RIG-I/MDA5, which in turn inhibited IFN-β production (Ding et al., 2017).

Furthermore, the different studies reported that SARS-CoV-2 N protein could induce inflammatory responses. SARS-CoV-2 N protein promotes the activation of NF-κB signaling by recruiting TAK1 and IKK complex, the key kinases of NF-κB signaling (Wu Y. et al., 2021). Another article reported that SARS-CoV-2 N protein associates directly with NLRP3 protein, enhancing the interaction between NLRP3 and ASC and promoting NLRP3 inflammasome activation to induce hyper inflammation (Pan et al., 2021). Taken together, SARS-CoV-2 N protein could inhibit IFN production and then prevent host innate immunity system from recognizing and combating infection in the first stages. When the host innate immune system sensed a virus, SARS-CoV-2 N protein could promote NLRP3 inflammasome activation, exuberant chemokines, and inflammatory cytokine production and flood the bloodstream faster than normal, resulting in a cytokine storm and disseminated damage to the host. It is consistent with the previous clinical results that low levels of types I and III interferons in the serum of patients with COVID-19 juxtaposed to elevated amounts of chemokines and proinflammatory cytokines (Arunachalam et al., 2020; Blanco-Melo et al., 2020; Huang et al., 2020).

## Other Evasion Strategies

### Preventing Host Protein Translation

Coronaviruses, as obligate intracellular parasites, hijack host cell components for viral translation and assembly by disrupting host mRNA splicing, translation, and protein trafficking to allow the translation of viral mRNA and concomitantly suppress host antiviral immune responses (Finkel et al., 2021). CoV NSP1 is a major viral virulence factor, which represses multiple steps of host protein expression. NSP1 of α*-* and β-CoVs, regardless of their low sequence identity, show similar biological function in inducing endonucleolytic cleavage of host mRNAs but viral mRNAs and inhibition of host translation (Nakagawa and Makino, 2021). SARS-CoV-2 NSP1 associates with the host mRNA export receptor heterodimer NXF1-NXT1, preventing proper interaction of NXF1 with mRNA export adaptors. As a result, many cellular mRNAs are withheld in the nucleus and cannot evade from the nucleus, and the infected cells do not release lots of IFN, alerting the immune system (Zhang K. et al., 2021). Besides, SARS-CoV-2 NSP1 binds to 18S ribosomal RNA in the mRNA entry channel of the 40S ribosomal subunit, and structural analysis by cryo-electron microscopy of *in vitro*-reconstituted nsp1-40S and various native nsp1-40S and nsp1-80S complexes revealed that the nsp1 C terminus binds to and obstructs the mRNA entry tunnel (Schubert et al., 2020; Thoms et al., 2020).

Furthermore, the C-terminal domain of NSP1 is located at the entrance of ribosomal mRNA, which prevents the entry of some but not all of the mRNAs. Its N-terminal can stabilize the binding between its C-terminal and ribosome acting as a non-specific barrier to block the mRNA channel, thus exerting a more thorough blocking effect and abrogating host mRNA translation (Zhao et al., 2021). Similarly, SARS-CoV NSP1 localizes exclusively in the cytoplasm and associates with the 40S ribosome to block the assembly of the translationally competent ribosome (Banerjee et al., 2020). Another study reported that SARS-CoV NSP1 prevents mRNA nuclear export by disrupting localization of nuclear pore complex protein 93 (Nup93) and altering the composition of the nuclear pore complex (NPC), thus subsequently suppressing protein synthesis (Gomez et al., 2019). MERS-CoV NSP1, distributed in the nucleus and the cytoplasm, selectively targets nuclear-transcribed host mRNAs for suppression, but mRNAs of cytoplasmic origin (Lokugamage et al., 2015).

Furthermore, SARS-CoV and MERS-CoV evade the NSP1-mediated translational inhibition by inducing a specific association between SL1 in the 5′ UTR of viral RNA and NSP1 (Terada et al., 2017). In brief, human HP-hCoV NSP1 efficiently disturb the cellular translation machinery. In addition, SARS-CoV-2 NSP16 binds mRNA recognition domains of the spliceosome’s U1 and U2 RNA components and disrupts mRNA splicing. SARS-CoV-2 NSP14 can shut down host protein translation, and the formation of the NSP14-NSP10 complex enhances translation inhibition executed by NSP14, suppressing host protein synthesis. This role of NSP14-mediated translation inhibition is conservative among three highly pathogenic *Betacoronaviruses* (Hsu et al., 2021). SARS-CoV-2 NSP8 and NSP9 interfere with host protein trafficking to the cell membrane by binding to discrete regions on the 7SL RNA component of the signal recognition particle (SRP) (Banerjee et al., 2020; Table 3).

**TABLE 3:** Other innate immune evasion strategies employed by HP-hCoVs.

**Protein**	**Virus**	**Mechanism**	**References**
**Preventing host protein translation**			
NSP1	SARS-CoV-2	Associating with NXF1-NXT1 and preventing proper interaction of NXF1 with mRNA export adaptors.	Zhang K. et al., 2021
	HP-hCoVs	Associating with the 40S ribosome	Lokugamage et al., 2015; Schubert et al., 2020; Thoms et al., 2020
NSP8 and NSP9	SARS-CoV-2	Interfering with protein trafficking	Banerjee et al., 2020
NSP16	SARS-CoV-2	Disrupting mRNA splicing	Banerjee et al., 2020
NSP14	HP-hCoVs	Shutdown the protein translation	Hsu et al., 2021
**Hijack of the antigen-presenting cells**			
Sike protein	SARS-CoV-2, SARS-CoV	Binding to DC-SIGN receptor on DC	Yang et al., 2004; Amraie et al., 2020; Campana et al., 2020
Virus	MERS-CoV	Infecting DCs productively	Chu et al., 2014
ORF8	SARS-CoV-2	Interacting with MHC-I directly and disrupting antigen presentation	Zhang Y. et al., 2021

### Hijack of the Antigen-Presenting Cells

Dendritic cells (DCs), the most potent antigen-presenting cells (APCs), are capable of entering peripheral tissues, taking up antigens, migrating to lymphoid tissues, and activating helper T cells (Anselmi et al., 2020). DCs link the innate and the adaptive immunity and can serve as an important target of viral replication and a vehicle for dissemination. However, the virus could exploit the function of DCs to evade from immune surveillance and facilitate cell-to-cell dissemination. Recent research reported that DC-SIGN serves as receptor, mediating SARS-CoV-2 entry into human target cells and leading to hypercoagulability, inflammatory response, and multiorgan dysfunction (Amraie et al., 2020; Campana et al., 2020). SARS-CoV spike protein could bind DC-SIGN receptor on DCs. Although SARS-CoV could not infect DCs, DCs can take up SARS-CoV and transfer it to susceptible target cells, which is useful for the viruses to break through the epithelial barrier and evade antiviral immune responses (Yang et al., 2004). MERS-CoV could productively infect DCs, leading to produce a large number of cytokines and chemokines and accelerating viral replication and dissemination (Chu et al., 2014). SARS-CoV-2 ORF8 directly interacts with major histocompatibility complex I (MHC-I) and disrupts antigen presentation for immune activation by downregulating MHC-I expression on the surface of cells, and therefore may help in immune evasion (Zhang Y. et al., 2021). However, SARS-CoV ORF8 does not affect MHC-I downregulation (Zhang Y. et al., 2021). Taken together, the SARS-CoV-2 adopts various strategies to evade the host innate immunity and the acquired immune response (humoral immunity and cellular immunity). Recent research reported that immune-compromised individuals with prolonged viral replication occurred a broader range of viral evolution (Kemp et al., 2020; Stanevich et al., 2021; Williamson et al., 2021; Table 3).

## Cytokine Storm Syndrome Induced by Highly Pathogenic Human Coronaviruses

In the initial stage of HP-hCoV infection, the virus evades the host’s innate immune response by using diverse strategies to obtain effective replication and establish a window of opportunity for infection. When the immune system detects the virus, the virus titer has reached a high level, which induces highly concentrated and prolonged proinflammatory cytokines and chemokines (IL-2, IL-10, GSCF, IP10, MCP-1, TNF-α, etc.) known as “cytokine storm” (Huang et al., 2005; Zhou et al., 2014; Fan et al., 2020). Cytokine storm is the uncontrolled release, fast-developing, and life-threatening, which can accelerate the depletion and exhaustion of T cells, leading to complicated medical symptoms such as fever, septic shock, capillary leak syndrome, acute respiratory distress syndrome (ARDS), disseminated intravascular coagulation, and multiple organ failure, and ultimately death in the most severe cases (Song et al., 2020). Early administration of IFN could help to reduce viral load and rarely induce cytokine storm, thus ameliorating the patient clinical feature. By comparison, delayed administration of IFN did not provide any advantage compared to placebo controls (Channappanavar and Perlman, 2017). Cytokine storm is responsible for the deterioration of HP-hCoV patients. Therefore, besides controlling viral load with early administration of IFN, the timing of attenuating cytokine storm could be life-saving. The current interventions for cytokine storm include traditional anti-inflammatory drugs (corticosteroids, chloroquine, and colchicines) and intravenous immunoglobulin, traditional Chinese medicine, and corticosteroids (Yang et al., 2021). However, the clinical treatment of cytokine storms has been proved challenging. It urgently needs a safe and effective drug to tailor treatment of cytokine storm at specific disease stages, ultimately ameliorating disease severity and return to homeostasis. In addition, not all HP-hCoV patients develop the same symptoms, but the immunological determinants of a poor prognosis are unknown, which remains to be explored further.

## Conclusion

In recent years, great progress has been achieved in studying the mechanism of the immune evasion of viruses. However, there are still many basic problems remaining to be solved. Most of the studies were conducted with overexpression experiments in cellular models. The experimental results drawn with different research methods are often inconsistent. This phenomenon is probably due to the difference in expression levels and phases of viral proteins expressed by plasmids and real viruses. Moreover, experimental results may be affected by different experimental systems, including protein tags and plasmid backbones. Validations should be conducted in multiple systems, especially under viral replication and even by animal physiological experiments.

When the worldwide COVID-19 pandemic emerged, vaccines and drugs were expected to end the pandemic. However, breakthrough infections occur even after vaccination because SARS-CoV-2 constantly mutates under various selective pressures, either naturally acquired post-exposure or vaccine acquired immunity, mainly due to the RNA polymerase lacking proofreading capability of the virus. The key mutant viral strains, including Alpha (first appeared in the United Kingdom), Beta (first appeared in South Africa), Gamma, and Delta (first appeared in Brazil), have been sweeping the world. Before the Delta strain was lifted, the Lambda strain has emerged. At present, asymptomatic infection allows the virus to continue spreading and mutating, making it more difficult to prevent and control the epidemic. The longer SARS-CoV-2 circulates, the more mutant strains emerge, which would challenge the current drug or vaccine. The changes of mutant strains and mutant genes of SARS-CoV-2 related to escaping host immunity should be continuously tracked and analyzed.

## Author Contributions

All authors contributed to the work. J-YL designed and wrote the manuscript. Z-JZ and QW helped with the figures. X-YG, YQ, Q-NH, and M-YZ revised the whole manuscript. All authors approved the final manuscript.

## Conflict of Interest

The authors declare that the research was conducted in the absence of any commercial or financial relationships that could be construed as a potential conflict of interest.

## Publisher’s Note

All claims expressed in this article are solely those of the authors and do not necessarily represent those of their affiliated organizations, or those of the publisher, the editors and the reviewers. Any product that may be evaluated in this article, or claim that may be made by its manufacturer, is not guaranteed or endorsed by the publisher.
